# How is leadership experienced in joy-of-life-nursing-homes compared to ordinary nursing homes: a qualitative study

**DOI:** 10.1186/s12912-022-00850-0

**Published:** 2022-03-29

**Authors:** Beate André, Frode F. Jacobsen, Gørill Haugan

**Affiliations:** 1 Department of Public Health and Nursing, Norwegian University of Science and Technology (NTNU), 7491 Trondheim, Norway; 2 NTNU Center for Health Promotion Research, 7491 Trondheim, Norway; 3 Nord University, Faculty of Nursing and Health Science, Levanger, Norway; 4 Centre for Care Research, Western Norway University of Applied Sciences, Bergen, Norway; 5 VID Specialized University, Diakonveien 12-18, 0370 Oslo, Norway; 6 Institute for Health and Care Sciences, Western Norway University of Applied Sciences, Bergen, Norway; 7 VID Specialized University, Bergen, Norway

**Keywords:** Nursing homes, Leadership, Qualitative study, Joy of life in nursing homes

## Abstract

**Background:**

Nursing homes are under strong pressure to provide good care to the patients. In Norway, municipalities have applied the ‘Joy-of-Life-Nursing-Homes’ (JoLNH) strategy which is based on a health-promoting approach building on the older persons’ resources. Meanwhile job satisfaction is closely related to less intention to leave, less turnover and reduced sick leave. The knowledge about adjustable influences related with job satisfaction might help nursing home leaders to minimize turnover and preserve high quality of care. This study explores leadership in Norwegian nursing homes with and without implementation of JoLNH: How does leadership influence the work environment and how is leadership experienced in JoLNH compared to ordinary Nursing Homes?

**Method:**

We used a qualitative approach and interviewed 19 health care personnel working in nursing homes in two Norwegian municipalities. The analysis was conducted following Kvale’s approach to qualitative analysis.

**Results:**

The main categories after the data condensing were [1] the importance of leadership, and [2] the importance of leadership for the work environment in a municipality with (a) and without (b) an implementation of the JoLNH strategy.

**Conclusions:**

The health care personnel in the municipality with an implementation of JoLNH emphasize that the leader’s influence may lead to increased motivation among the staff and better control of changes and implementation processes. Our findings may indicate that the employee from a JoLNH municipality experience a more trustful relationship to the leader.

## Background

In nursing home (NH) facilities, both the amount of care reliant older individuals and the request for qualified nursing care are rising due to demographic changes; the number of those 90+ will grow rapidly and the number of sick and frail elderly individuals in need of full-time care will rise accordingly [1–6]. However, significant yearly turnover rates and high sick leave among health care professionals in NHs lead to reduced availability of nurses and reduced staff continuity [7–9]. Meanwhile, job satisfaction is closely related to less intention to leave and lesser turnover [3, 10, 11]. Hence, knowledge about how to enhance health professionals’ job satisfaction might help NH leaders to minimize turnover, sick leave and preserve high quality of care [4, 9]. High job satisfaction in NHs associates with the prospect to deliver high-quality, person-centered care [12–14], effective leadership [4, 15], teamwork [16], and patient satisfaction [8, 17]. Low job satisfaction relates to lack of skilled employees [3, 4], insufficient leadership and authoritative leadership [4, 18], absence of collaboration [19], decrease of wellbeing, and nonappearance due to sickness [13, 20].

In an organization such as a nursing home, the work culture is a decisive factor in whether the facility achieves its goals. A healthy work environment involves that the employees feel secure, encouraged, and rewarded. Encouraging empowerment, commitment, and personal connections at work are critical in realizing a healthy work environment and quality patient care. Healthy work environments and nurse leadership are equally codependent. Nursing leaders are important in creating healthy work environments, patient care quality, and nurse job performance [21]. Nursing leaders’ capability has shown to correlate significantly with nurses’ experiences of the work environments [22, 23].

In Norway, where approximately 0,9 mill people are 65 years and older, almost 40.000 people live in NHs [24]. Hence, quality of NH care influences a great number of people. Therefore, the strategy ‘Joy-of-Life-Nursing-Homes’ (JoLNH) was established: the Norwegian government strongly endorses introduction of the JoLNH authorization program to the municipality health services [25]. The JoLNH strategy contains among others, tools for care quality and improved leadership. The JoLNH nursing care builds on a thorough mapping of each resident, individual calendar plans and specific procedures and routines as part of the organizations’ internal control [26]. In this way, a person–centered approach involving increased user participation is aimed for, and aspects such as regulations for management, quality improvement in the health care service, the dignity guarantee and the quality regulations are sought to be fulfilled [5, 27]. To become an approved JoLNH, nine claims must be realized [5]: [1] knowledge about the JoLNH philosophy and its implications, [2] a requirement to collaborate with schools, kindergartens and other organizations, [3] outdoors appreciating fresh air for all residents, [4] contact with animals, [5] permit the residents to maintain their hobbies, [6] musical and cultural activities, [7] pleasant atmosphere during mealtimes, [8] respectful communication with family and next of kin, and lastly [9] enable that seasons are highlighted in the NH environment [26]. The Joy-of-Life (JoL) foundation [26] conducts the JoLNH implementation including the authorization program together with the NHs and the municipality. In Norway, the term ‘municipality’ represents a geographical, political, and administrative units within the public administration in the state; there are 356 municipalities of different sizes and populations in Norway. When implementing the JoLNH approach, the municipality and the solitary NHs must fulfill the following: Make the NH prepared for the authorization process by satisfying the nine requirements, − the municipalities must pay a fee to the JoL foundation for the 1-year certification program as well as the recertification processes, − the municipality employs 2–3 JoL-coordinators who assist the NHs in the authorization and reauthorization processes and yearly the NH must work through a reauthorization process [5, 26, 28, 29].

Leadership in NHs has been characterized by unclear expectations, little inspiration to take initiative and scarce backing [30]. Caring relations among nurses and managers in NHs were studied showing that the nurses preferred caring manager performances such as: manager communication and feedback, manager competence and manager control [20, 31]. Substantial links among constructive leadership performances and increased patient satisfaction have been found [32], suggesting that leadership practice is important in realizing improved patient outcomes. Research has revealed that relation-oriented leadership, which endorses collaboration among NH residents and health care personnel, seems to increase empowerment of both residents and staff and may contribute to increased quality of care [33]. Leadership involvements influencing on how individuals relate to each other, their ways of communication, as well as better participation and relation-oriented leadership, can positively affect work culture and problem solving [34]. Communication and teamwork have been related to nurses actively partaking in their work culture and the prevention of turn over [35, 36]. Including health care personnel as an active part in decision-making and in creating a new and more relation-oriented leadership style appears to be critical to improve patient satisfaction in NHs [8]. Nurse managers and hospital administrators must use strategies that help nurses to manage job stressors effectively, communication and stress management, to better manage stressful situations and improve the work environment [37, 38].

Summing up, this literature review shows that nurses’ job satisfaction strongly relates to leadership, and that leadership, job satisfaction, turnover and sick leave relates to patients’ well-being and satisfaction. This study explores how an implementation of JoLNH influences on the healthcare personals experience with leadership and how leadership influences on the work environment.

Therefore, this study investigates the following research questions:

- How is leadership experienced in Norwegian NHs in a municipality with and without an implementation of JoLNH, respectively?
- How does leadership in Norwegian NHs influence on the work environment in a municipality with and without an implementation of JoLNH, respectively?

## Method

### Sample

Throughout winter 2019 health care personnel in NHs situated in two big Norwegian urban municipalities (one had implemented the JoLNH strategy, and one had not), were requested to contribute to a qualitative research interview. Two municipalities comprising a total of 84 NHs and 4200 beds were included; five NHs from each of the two municipalities were selected randomly; from these 10 NHs, 19 health care professionals participated in this study. The inclusion criteria for health professionals from JOLNHs was that they had been employed in the NH prior to, through and following the implementation of the JoLNH strategy. Concerning health professionals from ordinary NHs, the inclusion criteria was being employed at the NH minimum 1 year. The interviews were conducted at the respective NHs during the participants’ working hours, in a place guaranteeing no interruption.

The NH leadership provided the researchers with a list of potential study participants and their contact information. All potential study participants received written information about the study by email from the researchers, and 19 health professionals from 10 NHs volunteered to participate. The sample (*N* = 19) comprised 7 registered nurses, 8 licensed practical nurses, 2 occupational therapists, 1 social worker and 1 social educator, 9 study participants from ordinary NHs and 10 from NHs with an implementation of JoLNH. The study participants work experience in NHs ranged from 3 to 40 years, with a mean of 20 years. In total, 17 out of the 19 study participants were speaking Norwegian as their mother tongue and all 19 were female.

### Data collection

The individual interviews were held over a 6-month period and each interview lasted from 30 to 50 min. The interviewers used a semi-structured interview guide so that the study participants could talk spontaneously over the topic [39]. The study participants were asked question about; “To what extent can your leader influence on your desire to work at the NH for a long time to come?” and “What hinders and promotes a good working environment as you see it?”. Before conducting the interviews, the two interviewers underwent a short information gathering along with coaching to employ the same method. All interviews were audiotaped and transcribed precisely by skilled transcribers.

### Analyses

The transcribed interviews were systemized, and the content along with the meaningful units in these data were debated by the researchers using Kvale’s method for qualitative analysis [40]. To protect the confirmability of the results, two researchers were involved at all stages, the first and the last author [41]. The first and last author have long and extensive experience in the use of a qualitative method, both in publications together and separately [5, 42–45] and together with the second author [46, 47]. Dependability were inspected by using the five stages from Kvale’s method [40]: categorization of meaning, condensation of meaning, structuring of meaning, interpretation of meaning, and ad hoc methods for generating meaning [40, 41]. The categories appeared from the material; we interpreted the meanings of the findings, which we emphasized and condensed into dimensions with their unique arguments unbroken. Subsequently the results were condensed; we created narratives on every issue. In this procedure, the two researchers discussed the meaning of the statements to ensure context sensitive interpretation and to understanding the statements of sense of the total setting which the statements occurred [48]. Then the results were systematized, condensed, and sorted in preliminary categories using NVivo 12Pro [49].

### Ethical considerations

The ethical guidelines of voluntary participation, written informed consent and the possibility of withdrawal at any point were followed. The participants were informed about the purpose and aim of the study. The data were anonymized. The results are presented in a way ensuring the participants’ integrity and anonymity: for this reason, the individual participants’ professional background is not included. The Norwegian Centre for Research Data, Data Protection Services, was notified of the project (ref.nr. 238,331). Prior to this, an application was sent to the Regional Committee for Medical and Health Research Ethics, who declared that their approval for the current project was not required according to the Norwegian Health Research Act.

## Results

The results are showing two main categories: [1] “The importance of leadership”, with “duties of a good leader” and “positive leadership” as sub-categories and [2] “leaders’ importance for the work environment”, and “to work and solve tasks together” as subcategory. The findings are further elaborated on presenting some central statements by the study participants, separated in two groups: (a) study participants from municipalities with an implementation of JoLNH and (b) study participants from a municipality with ordinary NHs. The study participants are numbered in parentheses at the end of each statement, and they are described as study participants or employees. 

### The importance of leadership

All study participants emphasize the importance of the leadership to provide a high quality of care to the NH patients.

#### With JoLNH implementation

The study participants stated that their personal relationship with the leader was important, as well as how they feel that they are handled by the leader. A typical statement was:

*“That you get through with the ideas you have - that it is a responsive leadership and that we work together and respect each other” (a2).*

Most of the study participants emphasized that the leader’s behavior and actions have an influence on them: behavior such as good communication, respect, and being open were underlined. Also, the availability of the leader and that they had confidence in the leader was commented on. Furthermore, how the health professionals could work together to increase quality of the patient care was highlighted by the study participants as important.

#### Ordinary NHs

These study participants described leadership with some of the same characteristics as the study participants in the municipality with an implementation of JoLNH, and some were different, one statement was:

*“She is caring and able to accept and receive criticism”,* (b5).

These study participants focused on problem solving in collaboration with the leader, good collaboration, and the importance of stability among the leaders. Some of the study participants also concentrated on both the care aspect and the ability to receive and accept criticism as an important consideration for a leader. It was mentioned that the leader said that she was an important employee: this message has been significant for her.

### Duties of a good leader

These statements focused on expectations and preferences of how a leader should act toward the employees. By means of his/her behavior, the leader facilitates that the employees can feel good in their workplace, as well as competent in their work.

#### With JoLNH implementation

The statements in the JoLNH address how the leader should act and behave toward the employee, and how the leader may assist the employees in achieving their goals, as said:

*“I am motivated by the fact that they have faith in us - that they support us 100%”,* (a6).

Study participants also commented on the fact that the leader should give both positive and negative feedback to the healthcare workers but expressed the importance of taking both praising and criticism to the person of concern. Several of the study participants stated that the leader must help with extra difficult cases and support them when they supposed to take difficult decisions.

#### Ordinary NHs

In the non-JoLNH municipality, the study participants underscore that the leader understood the needs of the employees and that the manager’s responsibility is to be the employees’ external voice, e.g., in case of too scarce resources, as one stated:

*“A leader must be far-sighted and bold - have courage – both closures and new construction have been discussed”,* (b6).

The non-JoLNH municipality has also done some changes and it seems to have been discussions related to external resources. These challenges may be difficult to handle, but it seems like the informants meant that the leaders had a responsibility to “fight” for the NH’s resources. One informant stated that her effort for the NH were decisive in the discussion with the leader, as stated:

*“Because of what I do, I get what I want when I want time off”,* (b7).

This seems like a reward system for healthcare workers at the NH, so if they perform satisfactorily, they will get time off when it is suitable for them.

### Positive leadership

This subcategory emphasizes the qualities characterizing a good leader and what is perceived as positive from the employees’ perspective.

#### With JoLNH implementation

The study participants depict how the leader had a positive role related to the patients, as well as the importance of the leader’s positive engagement in the JoLNH caring approach, as stated:

*“We have worked together in nursing care for the patients - she endures a lot”,* (a10).

The informants seem to give positive feedback to leader who participates bedside with them, caring for the patients. They also approve the leader who knows the patients’ names and engaged themselves in the day-to-day care. They also underscored the positive impact of a leader encouraging to changes.

#### Ordinary NHs

This study participants emphasized both the leader who makes jokes and works together with them, inspiring them, and the leader who is not afraid to make changes, as said:

“*A good leader thinks ahead and is not afraid to take on neither technology nor new research*”, (b1).

Study participants in ordinary NHs commented on the importance of the leader being positive to changes and to leaders who work together with them and participates bedside. The study participants state that their leader discusses with them how to do the work:

*“She asks, how do you want it - she is open to how I want to do things”,* (b2).

This study participant is positive to the leaders who asked and discussed with how they are used to solve tasks and not just take over or show how to perform the tasks.

### Leaders’ importance for the work environment

This category embraces how good or bad leadership influences on the work environment in the NHs.

#### With JoLNH implementation

The study participants emphasize the leader’s important role in facilitating a good work environment, as well as in implementing the JoLNH strategy:

*“The contact with the leader is very important, she is very good in communication and takes care of the good routine also when implementation changes* - *she has a great impact on the work environment and makes me still want to work here*”, (a8).

Here the study participants commented on how important the leader is for the work environment, and that both the leader’s ability to communicate and listen to ideas and respects the employees. Some study participants also state that it is important to have a leader who is in control, so everyone experience that it is the manager who decides, at the same time as the leader also listens to the employees. In times of high turnover, it is important to see how this may be affected:

*“If we get a little extra resource to implement things then our motivation to continue working here increases, which is good for the work environment”,* (a2).

The study participants emphasized the importance of a leader who engage in getting extra resources for implementing positive interventions or changes; this affected the employee’s intention to keep working at the unit. A stable work force is good for the work environment.

#### Ordinary NHs

The study participants concentrate on several challenges that has led to negative outcomes for the work environment. It seems like working independently and doing things in their own way are issues, which the leader has not been able to sort out. The leader’s voice was not a strong voice among these study participants:

*“I work very independently and accomplish a lot of my own ideas - I think that’s good - implying positive changes for the environment”,* (b9).

It appears like the study participants have experienced some challenges in the work environment and that no one have been able to influence on the situation in a positive way, as stated:

*“When challenges with the patients occurs, we are not able to stand together – that is destructive for the work environment”, (b1).*

Dealing with conflicts both in connection with patient care and between the employees, is challenging for healthcare personnel. In case of conflicts and challenges leadership may be important:

*“I have mentioned that even if we do not have any good chemistry in private, we must be able to work together”* (b2).

The conflict between the employees, as they describe, does not seem to be tried to be resolved by a leader, but is given the opportunity to flourish and develop. It is left to themselves to find solutions.

### To work and solve tasks together

This subcategory involves different aspects of working together with the leader and the employees.

#### With JoLNH implementation

The study participants focus differently; among other things, they describe that the leader may give patients promises that are difficult to keep, which is not a sign of a good collaboration, as stated:

*“It is very important to keep agreements with patients and that the leaders, yes we have experienced that they can promise more than we can keep - they promise things that are impossible”,* (a1).

Furthermore, the study participants state the need for clarified roles, as mentioned:

*“Every nursing home is different, so it is important to clarify the different roles we have”,* (a8).

Solving tasks together, an open communication is important. Leaders and employees discuss together before promises are given and clarifying of roles and functions may be important to avoid tensions and misunderstandings.

#### Ordinary NHs

In this municipality, the study participants describe difficulties when working together. The work with patients is satisfying but working together with the leader and other employees are challenging, as stated:

*“It is a meaningful job, to be in contact with patients and leaders - but sometimes I would like to get time off from the rest of the staff”,* (b3).

It seems like conflicts between the employees affect their collaboration with the manager. Furthermore, the study participants demonstrate a positive will to collaborate with the leadership to get more resources. They appreciated a leader who joined them in achieving resources to provide good NH care.

## Discussion

This study explores two research questions: [1] how is leadership experienced in Norwegian NHs in a municipality with and without an implementation of JoLNH? and [2] how leadership influences on the work environment in a municipality with and without an implementation of JoLNH?

### How is leadership experienced in Norwegian nursing homes with an implementation of JoLNH?

The study participants working in certified JoLNHs stated that the JoLNH implementation influenced on their intention to stay. In light of the increasing number of elderly with an expected need for NH care in the years to come, this is an important finding [1–5]; finding ways to strengthening NH employees’ intention to stay and thereby decrease turnover are important aspects to increase care quality and patient safety in NHs [4, 8]. Study participants from the JoLNH-municipality state that they collaborate very well with the leader and that this collaboration is positive for the work environment. Collaboration has been associated with attachment to the organization [37, 50, 51]. Nursing leadership influences positively on the work environment by facilitating positive values such as collaboration, and work satisfaction [37, 52]. To strengthen the work environment, the present findings suggest that the leader must encourage more collaborative behavior than individuality. Study participants from a municipality with an implementation of JoLNH stated that their leader trusted them and focused on motivation. Even if the study participants from the JoLNH municipality also comment on negative issues, it seems like their focus is not on negative aspects. Also, when describing the duties of a leader the study participants from a JoLNH municipality seem to emphasis the help they receive from the leader, and that they appreciate both positive and negative feedback. This may indicate a more trustful relationship to the leader.

Moreover, the study participants from the JoLNH municipality centered more on how a leader’s influence may lead to increased motivation, intention to stay and having planned changes and implementations under control. Successful implementation depends on planned changes in the work environment, relationships, and skills [53, 54]. Therefore, paying attention to the work environment during an implementation may lead to a more successful implementation based in involvement and engagement by the employees.

### How is leadership experienced in Norwegian nursing homes without an implementation of JoLNH?

The study participants from ordinary NHs describe a work environment with many challenges. Difficulties with taking decisions, problems with working together and disloyalty. They also described more discussions and conflicts, both related to the administration of the municipality an, the leader at the unit and between the employees. The study participants also described a kind of reward system for getting benefits if they performed adequately. Even if the study participants described this, it may have been an expression of how they perceive the situation, and that it is unclear how benefits such as when one is given time off are distributed.

The study participants from the municipality without an implementation of JoLNH described most negative aspects of the leaders’ influence on the work environment, and negative impact by the leader’s engagement. The study participants also described a work environment with personal conflicts and no one having the responsibility to solve them. Some informants described that they had made efforts to solve conflicts; they laced a clear voice from a leader. Nurses’ perception of the work environment relates significantly with nursing leaders’ expertise to lead [22, 23]. This might imply that a stronger focus on leadership in this municipality may be necessary. The work at the NH were experienced as positive and meaningful, however the high level of conflicts was challenging and demotivating. Strong links among positive leadership performances and improved patient care have been found [32], suggesting that leadership performance is important in achieving increased patient results, and good quality care are linked to better staff motivation [4].

### Summarize of differences and similarities between NH’s with or without JoLNH

To sum up, the present results show that employees in NHs emphasize their relationship with the leader as crucial to their motivation and thriving; they are dependent of feedback from their leader and need to feel approved and valued. What’s more, they need a leader who is clear, consistent, honest, and loyal toward both patients and staff. There was a difference between NHs with and without an implementation both in relation to the working environment and in relation to the leader’s involving in the working environment. In ordinary nursing homes, it was revealed that they had conflicts that were allowed to unfold freely without the leaders intervening. In nursing homes with an implementation of JoLNH it emerged that the employees together with the leaders tried to find solutions to challenges. Earlier studies have found that leadership concerns influencing on how people interact, their habits of communication, as well as better involvement and relation-oriented leadership, can definitely impact on the work environment and finding resolution to challenges [34].

Research has established a link between constructive leadership performances and increased patient approval [4, 8, 32, 46], suggesting that leadership practice is important to realize improved patient outcomes. Several study participants stated that their relation to the leader was important for their motivation in their work. They were dependent of getting feedback from the leaders, and if their work was appreciated, they described a feeling of being approved and respected. The present study participants emphasized open communication and working together with the leader. Furthermore, having fun together and a light tone and that the leader showed faith in the staff were central. It is easier to discuss significant issues with each other if the atmosphere is respectful, trusting, open, and inclusive. This corresponds with a former study, where use of humor and other behaviors that may reduce some pressure away from the health care personnel were highlighted [4]. The leader’s responsibility for the atmosphere in the NH has been commented on before, showing that the characteristics of the nursing leadership set the standard of practice and tone in the environment [37].

Previous research has focused on the importance of a healthy work environment to promote engaged communication, prevent turnover and intention to leave, and thereby increase care quality and patient safety [37, 55, 56]. Differences were found between those NHs who had an implementation of JoLNH and those who did not have it in relation to the work environment. In the ordinary NHs, the work environment was described as characterized by conflicts, some experienced that they received a form of reward if they behaved in a special way, others thought colleagues were a burden. Leadership is an important factor in development of a good work environment. Relationship, teamwork and strong group relation were related to work culture in five of the articles presented in another study [8]. The importance of giving empowerment to the healthcare personnel working in nursing homes is both an organizational and an interpersonal issue. Being given empowerment and influence over their own work situation, the healthcare workers can be more committed and involved in the goal of obtaining best possible care to the residents [8].

The present findings illustrate the importance of leaders to show up frequently in the ward partaking in the everyday work; this goes for NHs both with and without an implementation of JoLNH. Moreover, the significance of the leader taking part in changes and using new knowledge was emphasized. Improving health professionals’ participating in decision-making, increasing empowerment and influence seem to be crucial factors to improve quality of care [8]. The present study participants express a positive attitude toward changes and the use of new knowledge; such positive attitudes are important to empower NH employees [4]. Development of new interventions and using new knowledge may contribute to improved quality of care, which is important for thriving and meaningfulness among the staff [4, 34, 46]. Discussions about roles and role perceptions are important in achieving a good and healthy work environment [21]; open communication and common problem solving [18, 19] are important to facilitate job satisfaction among employees. The role of the leader is important to achieve both higher job satisfaction and increased patient care quality [4, 18]. The JoLNH strategy aims to increase quality of care and leadership [26]; the present findings indicate that the JOLNH strategy was beneficial to the work environment, work satisfaction and positive leadership.

### Limitations

The present data were translated from Norwegian to English; when translating data, it is always a risk to misinterpret and mislay some of the unique meaning. This is a qualitative study, with qualitative interviews, so it is the informant’s understandings constitute the basis of the present findings. There were no men in the sample who were interviewed, and it is conceivable that other nuances would have emerged if the sample had included both genders. This study was conducted in Norway with a Norwegian population. In Norway, work conditions are relatively favorable for workers in an international comparative perspective, so the results of this research cannot be generalizable to other contexts without taking this into consideration [57]. The present findings may, give an indication as to the direction that research ought to follow in subsequent studies.

## Conclusion

The present results show that health professionals in NHs emphasize their relationship with the leader as crucial to their motivation and thriving; they are dependent of feedback from their leader and need to feel approved and valued. What’s more, they need a leader who is clear, consistent, honest, and loyal toward both patients and staff. Open communication facilitating an atmosphere of mutuality, showing faith, and valuing each other, a light tone, being present and available characterize good leadership in the NHs represented in this study. The study participants in the JoLNH municipality were more concerned about how a leader’s influence may lead to increased motivation and intention to stay, as well as having planned changes and implementations in hand. It seems like taking part in the implementation of JoLNH lead to more positive attitudes toward changes and implementations. Furthermore, the employees were more concerned about collaborations with another employee and the leader. Our findings indicate that the employee from a JoLNH municipality experience a more trustful relationship to the leader.

## Data Availability

The datasets used and/or analyzed during the current study available from the corresponding author on reasonable request. We confirm that all methods were performed in accordance with the relevant guidelines and regulations.

## Abbreviations

NH: Nursing homes
QoL: Quality of life
JoLNH: Joy of Life Nursing Homes
JoL: Joy of Life
